# Modern Sociology

**Published:** 1901-08-31

**Authors:** 


### Modern Sociology.

### THE COLONIES AND THE CHILD.

"Methods of dealing with State Children in Australasia" was the subject of a paper recently read by Miss Hilda Martindale. The reading took place at 10 Lancaster Gate, W., the residence of Mr. Albert Spicer, M.P.; and the audience was composed of Poor Law Guardians and others interested or directly concerned in the care of children.

After a careful inquiry made during a recent visit into the various methods adopted by the Australasian Colonies, Miss Martindale stated that South Australia was in the van of progress for three reasons: (1) the absence of large schools; (2) and of workhouse contamination; (3) the systematic control of what are technically called neglected children. Two women were largely instrumental in bringing about this condition of things, viz., Miss Clarke and Miss Catherine Spence. The provisions of the State Children's Act of 1895 were carried out by the State Children's Council, composed of both men and women. A State child was defined as destitute, neglected or convicted; and the Council had (1) the care of all State children; (2) power to license foster-mothers for children under two years of age; (3) to grant licences for maternity homes and to supervise them; (4) to appoint and control institutions for State children; and (5) the control and administration of all property voted and acquired for the purposes of the Act. The State child was first taken to the receiving depôt, which was under the charge of a matron; it was then tried by the magistrate, not in a police court, but in a "place" which might be the office of the Council, and the trial might be quite private if the magistrate saw fit. From the depôt the child was either boarded out or sent to a special school or reformatory; and to the depôt he or she might return up to the age of eighteen years, when the period of State childhood ceased. Boarding out was so extensively practised that out of the 1,248 State children dealt with in the report for 1900 over 1,000 were provided for in this way, while a comparatively small number were sent to reformatories. Two homes had just been opened for special cases difficult to deal with, and whenever possible girls and boys were placed out in situations, though still under the control and supervision of the Council. Miss Martindale added that South Australia was in advance of other colonies in having a separate court for children; and that it was the only place which had an entirely separate lock-up system. During the period awaiting trial one of the officers endeavoured to become acquainted with the child's history, and she never fully realised the importance of this until she visited Chicago. Two other lines of progress were the systematically organised collection of maintenance fees from parents or relatives, and the completeness of legislation.

The colony of Victoria was next dealt with, and here the experiment of boarding out young mothers with their babies was touched upon. As far as could yet be seen, it seemed likely to prove very beneficial to both mothers and children, and it would be watched with the greatest interest. In this colony, too, Miss Martindale was much pleased with the Salvation Army Homes, towards which she ackowledged having entertained some prejudice. In each of the nine visited the same spirit of love seemed to be the ruling principle. Mrs. Booth, who had the control of them, believed greatly in pretty things and their effect on character; the children attended church or chapel in the village; one of the five or six officers was always a trained teacher; and these officers as a class were distinctly superior to what she had expected to find, being only appointed after careful selection. The officers slept and ate with the children, who made their own clothes, worked in the garden, &c. Punishments were seldom resorted to. An instance occurred to her in which severe treatment would probably have had an injurious effect. A girl came to one of these homes whose mother had been killed and whose relatives had committed suicide; she was a very difficult child to manage, and only after careful inquiry was it discovered that she was fretting about a little brother and sister whom her mother had left in her care. They were, happily, found, and, her mind being at ease, the girl gave no further trouble. The doors of these homes were never locked, and the children could run away if they wished. Few did so. There was a great work too in small reformatory homes, which had been entirely revolutionised during the last few years. This was owing to the offer, by Mrs. Rowe, to take five or six girls to live a free, out-of-door existence on her farm. The Government accepted her offer, sent her some girls, who were placed under the care of ladies really interested in them, and the rate of payment, beginning at 5s. per week for each, was afterwards raised to 10s. Boarding out was extensively practised.

Matters in New South Wales were complicated by the existence of two departments dealing with State children; and, as a result of the Act of 1896, two policies of boarding out. Miss Martindale dealt briefly with Tasmania and New Zealand; and summed up by saying that if children were to grow up to be self-respecting they must be treated as separate individuals; that, rightly administered, boarding out seemed to be the best method; and that special children could be best dealt with in small probationary homes. By these means the child of want, misery, and sin might become upright, moral, and a good citizen.

The discussion following the reading of the paper turned chiefly on the subject of boarding out. One of the guardians present thought the powers under the Local Government Board were too limited, while another thought they were ample if fully taken advantage of. In cost per head it was found that England and Australasia spent a nearly equal sum. Mrs. Mills Carver gave a description of one of the "scattered homes" at Bradford (Yorkshire), with which she had been much pleased, and Mr. Langham urged the claims of the State Children's Aid Association. He thought great improvements would be possible if it received more support from the Guardians. At Southwark from 100 to 150 children had been annually boarded out for some years past, and he thought that if other boards could only be imbued with the spirit which actuated the Southwark Guardians their example would be followed. A subsequent speaker was afraid his views of the progressiveness of guardians were a little too rosy.

In reply to a question, Miss Martindale said that although the children were committed until the age of eighteen, no obstacle was placed in the way of their returning to their parents, if it appeared advisable to allow them to do so.

